# Advances in Host-Free White Organic Light-Emitting Diodes Utilizing Thermally Activated Delayed Fluorescence: A Comprehensive Review

**DOI:** 10.3390/mi15060703

**Published:** 2024-05-26

**Authors:** Wenxin Zhang, Yaxin Li, Gang Zhang, Xiaotian Yang, Xi Chang, Guoliang Xing, He Dong, Jin Wang, Dandan Wang, Zhihong Mai, Xin Jiang

**Affiliations:** 1 College of Information Technology, Jilin Engineering Research Center of Optoelectronic Materials and Devices, Jilin Normal University, Siping 136000, China; zwx00719@163.com (W.Z.); 13944698385@163.com (Y.L.); cx02008@126.com (X.C.); donghe@jlnu.edu.cn (H.D.); 2 Key Laboratory of Functional Materials Physics and Chemistry of Ministry of Education, Jilin Normal University, Siping 136000, China; 3 Key Laboratory of Preparation and Applications of Environmental Friendly Material of the Ministry of Education, College of Chemistry, Jilin Normal University, Changchun 130103, China; hanyxt@163.com; 4 Jilin Special Equipment Inspection Center, Jilin Special Equipment Accident Investigation Service Center, No. 866 Huadan Street, Longtan District, Jilin 132013, China; guoliangxingjlsei@163.com; 5 Hubei Jiufengshan Laboratory, Wuhan 430206, China; wangdandan@jfslab.com.cn (D.W.); maizhihong@jfslab.com.cn (Z.M.); 6 State Key Laboratory of Integrated Optoelectronics, College of Electronic Science and Engineering, Jilin University, Changchun 130012, China; jiangxin22@mails.jlu.edu.cn

**Keywords:** WOLED, intrinsically host-free engineering, thermally activated delayed fluorescence (TADF), phosphorescence

## Abstract

The ever-growing prominence and widespread acceptance of organic light-emitting diodes (OLEDs), particularly those employing thermally activated delayed fluorescence (TADF), have firmly established them as formidable contenders in the field of lighting technology. TADF enables achieving a 100% utilization rate and efficient luminescence through reverse intersystem crossing (RISC). However, the effectiveness of TADF-OLEDs is influenced by their high current density and limited device lifetime, which result in a significant reduction in efficiency. This comprehensive review introduces the TADF mechanism and provides a detailed overview of recent advancements in the development of host-free white OLEDs (WOLEDs) utilizing TADF. This review specifically scrutinizes advancements from three distinct perspectives: TADF fluorescence, TADF phosphorescence and all-TADF materials in host-free WOLEDs. By presenting the latest research findings, this review contributes to the understanding of the current state of host-free WOLEDs, employing TADF and underscoring promising avenues for future investigations. It aims to serve as a valuable resource for newcomers seeking an entry point into the field as well as for established members of the WOLEDs community, offering them insightful perspectives on imminent advancements.

## 1. Introduction

In contemporary society, electronic displays have become an indispensable component of various industries. The growing demand for display devices has led scientists to explore new and efficient light-emitting materials and structures of devices. Among the proposed alternatives, organic light emitting diodes (OLEDs) have become the most promising display technology, experiencing rapid and dynamic development [1,2,3]. Compared with traditional LED materials, OLEDs have significant advantages, such as vibrant color reproduction, flexibility, ultrathin form factor, and low energy consumption. These characteristics make OLEDs a convincing innovative solution to meet the requirements of ultra-lightweight, ultrathin, energy-efficient, and high-color-saturation displays [4,5,6,7]. 

Various types of WOLEDs have been developed, including all-phosphorescent white light [8,9], all-fluorescent white light [10,11], TADF white light [12,13], hybrid white light [14,15,16], and others. The first generation of OLED luminescent materials, namely fluorescent materials, were unable to effectively utilize the generated triplet excitons, resulting in an internal quantum efficiency (IQE) [17] of only 25%. Therefore, devices using these materials have difficulty in achieving an external quantum efficiency (EQE) of over 5%.

In 1998, Forrest and Professor Ma Yuguang from Jilin University introduced the second generation of luminescent materials [18]: phosphorescent materials. In 2012, Adachi et al. discovered a class of organic materials capable of achieving 100% IQE by leveraging the phenomenon of TADF [19]. By reducing the energy gap between singlet and triplet excitons, triplet excitons can be converted to singlet excitons under the condition of thermal activation, resulting in radiative recombination [20]. Although fluorescent materials can achieve complete utilization of excitons, the commercial feasibility is still limited due to their high cost [21,22,23].

Organic luminescent materials can be categorized based on their distinct luminescent mechanisms, such as fluorescent materials [24,25], phosphorescent materials [26], TADF materials [27,28], and others. The exciton decay process of organic materials can be divided into radiative transition, intersystem crossing (ISC), reverse intersystem crossing (RISC), and internal conversion (IC). The triplet state of exciton transitions back to the excited singlet state through the anti-system crossing process, where the fluorescence generated by the deactivation transition to the ground state is called delayed fluorescence, and the T_1_ of TADF transforms into the luminescent S_1_ through RISC [29,30,31], thus achieving 100% theoretical quantum effect. High-quality white light emission can be achieved based on exciplex and electroplates emission [32,33].

**Figure 1:**
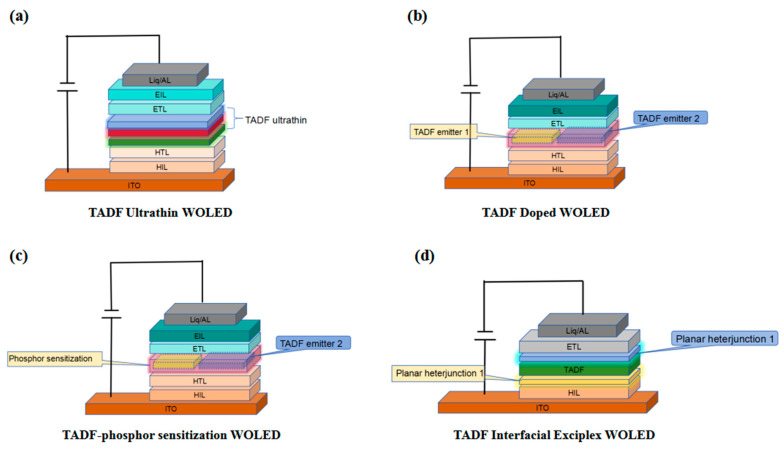
(**a**) TADF ultrathin WOLED. (**b**) TADF doped WOLED. (**c**) TADF-phosphor sensitization WOLED. (**d**) TADF-interfacial exciplex WOLED.

We listed four common types of TADF-WOLEDs, as shown in Figure 1, and summarized the key data from their representative literature in Table 1. Most research works concerning OLEDs are carried out based on doping and excitable compounds, but at the same time, there are problems such as complex device structure [34,35], strict doping ratio [36,37], and easy contamination during the evaporation process [38]. However, ultrathin WOLEDs (the luminous layer is usually a few nanometers or even less than 1nm of the device) can make it easier to conduct experiments by virtue of its simple device structure. Therefore, this work summarizes the research progress of host-free TADF-WOLEDs in terms of compound synthesis, device structure, and device photoelectric properties.

## 2. WOLEDs Based on Host-Free TADF-Conventional Fluorescence Materials

The pursuit of high electroluminescence efficiency in WOLEDs has led to extensive research on all-phosphorescent systems and fluorescent–phosphorescent systems. These systems utilize phosphorescent materials, which exhibit high efficiency in exciton utilization. However, the widespread application of WOLEDs has been hindered by the limited availability of strong blue phosphors [49,50,51]. In contrast, the traditional emitting layer of WOLEDs can only harness singlet excitons. When doped into TADF materials, the electroluminescence (EL) efficiency is significantly enhanced compared to common host materials [52]. Moreover, full-fluorescent WOLEDs utilizing TADF materials show outstanding competitiveness and advantages as hosts and sensitizers in terms of high colorimetric purity and the stability of fluorescent emitters. These advancements highlight the potential and desirability of full-fluorescent TADF-based WOLEDs over traditional full-fluorescent WOLEDs [53,54].

**Figure 2:**
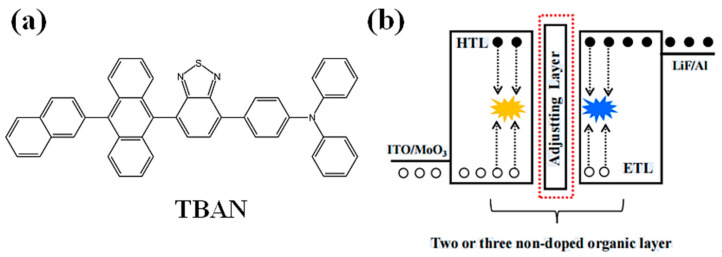
(**a**) Molecular formula and (**b**) structural formula representation.

In a recent study by Miao et al. in 2021 [55], a multifunctional compound incorporating tetra(4-bromophenyl)anthracene (TBAN) was designed and synthesized. Through the utilization of hybrid local electron and charge transmission mechanisms, as well as aggregation-induced luminescence, a novel and highly simplified two-color WOLED was proposed. The WOLED comprised solely three host-free organic emitting layers, with the molecular formula and structural diagram depicted in Figure 2. TBAN acts as the yellow emission layer and hole transport layer, while Bepp_2_ functions as the electron transport layer and a blue emission layer, ultimately producing white light. To achieve a more balanced white emission, a charge-regulating layer was incorporated between the two transport layers. This adjustment facilitated the controlled distribution of carriers and excitons in the transport layer, enhancing the overall performance and color balance of the WOLED.

In their experimental study, Miao et al. aimed to develop WOLEDs by combining the simultaneous emission of TBAN and bis[2-(2-pyridinyl)phenolato]berylliuM (Bepp_2_). Initially, the authors tested the EL performance of yellow and blue OLEDs to assess the potential for achieving white emission. The connection lines of the two best-performing devices’ Commission Internationale de l’Eclairage (CIE) coordinates intersected within the pure white light region, indicating the possibility of achieving high-quality white emission by balancing the intensity of TBAN and Bepp_2_ emissions [56,57]. Considering the significant influence of Bepp_2_ thickness on device performance, Miao et al. designed a series of two-color WOLEDs with varying Bepp_2_ thicknesses. The aim was to adjust the emission spectrum by optimizing the Bepp_2_ thickness. To balance the distribution of excitons in the yellow and blue emission transport layers, a high-level charge-regulating layer composed of TCTA was inserted between the two respective transport layers [58]. The three-layer host-free structure of TBAN/4,4′,4″-Tris(carbazol-9-yl)-triphenylamine(TCTA)/Bepp_2_ was optimized in WOLEDs, and 86 white-light devices with one of the highest CRI reported in the literature were obtained. Additionally, the maximum brightness achieved was measured at 43,460 cd/m². The EL spectrum’s change in CIE coordinates exhibited a minimal shift of (0.00, −0.01) when the turn-on voltage increased from 5 V to 7 V. This change corresponded to a brightness increase from 1011 cd/m² to 9310 cd/m^2^.

In a separate study conducted by Zhao et al. in 2023 [50], the all-fluorescence device consisted of an orange-light-emitting layer sandwiched between two sky-blue-light-emitting layers. The design achieves a record power efficiency (PE) of 130.7 lm/W and 31.1% EQE. For a WOLED composed of all-TADF, triplet-triplet and singlet-triplet annihilation would be more serious due to the longer lifetime of triplet excitons in TADF materials [31]. Therefore, the hybrid WOLED structure composed of TADF luminescent layers is very important as it can prevent excitons from reaching the non-radiative lowest triplet excited state of the traditional luminescent layer.

In 2018, Zhao et al. [59] reported a yellow TADF emitter with strong emission bis(2-(diphenylphosphine)phenyl)ether oxide (OPDPO), a blue emitter 9,9′-((2-(4′′-(9H-carbazol-9-yl)-[1,1′-biphenyl]-4-yl)ethene-1,1-dial)bis(4,1-phenylene))bis(9H-carbazole)(2CzTPEpCz) with high aggregation-induced emission (AIE)-active fluorescent materials in the T_1_ state. Similarly, in order to achieve the AIE effect, Wang et al. [60] added donor/acceptor pairs to the polymer to form a double space charge transfer channel. The white light device achieved a 14% EQE.

**Figure 3:**
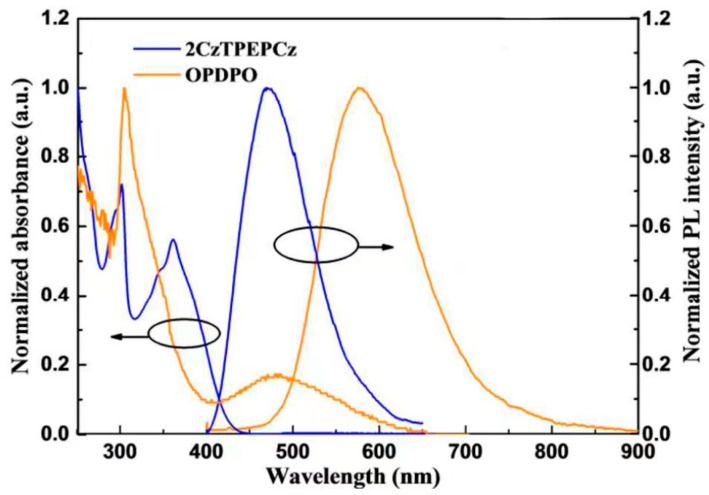
PL spectra and UV-vis absorption of OPDPO and 2CzTPEPCz films.

It is envisaged that a blue AIE-active fluorophore will be combined to realize completely host-free high-efficiency WOLEDs. As shown in Figure 3, the OPDPO and 2CzTPEpCz peaked at 577 nm and 470 nm, respectively. The S_1_ and T_1_ states of OPDPO were reached at 2.59 and 2.46 eV. Due to the small difference in the excitation energy between the two states, the non-emissive T_1_ state of 2CzTPEpCz is higher than that of OPDPO. When the two emissive layers (EMLs) simply stack together without any intermediate layer, the triplet exciton quenching of OPDPO can be effectively inhibited by 2CzTPEPCz.

Most reports on high-efficiency TADF devices for use in host-free OLEDs focus on blue and green emitters rather than yellow-light-emitting layers [61,62], and solution-treated OLEDs have the potential to be printed into complex structures and luminous regions [63,64]. Thus, Zhao et al. also prepared a host-free device for OPDPO: indium-tin-oxide (ITO)/PEDOT: PSS/4,4′-bis(9H-carbazol-9-yl)biphenyl (CBP) (33 nm)/OPDPO (7 nm)/1,3,5-tris(N-phenylbenzimidazol-2-yl)benzene (TPBI) (40 nm)/Mg: Ag. The maximum current efficiency (CE), PE, and EQE are 37.6 CD/A, 14.8 lm/W, and 16.6%, respectively. The maximum brightness is 3600 cd/m^2^, which has an EL performance suitable for practical application. Host-free devices based on 2CzTPEpCz: ITO/PEDOT: PSS (40 nm)/CBP (20 nm)/2CzTPEPCZ (15 nm)/TPBI (40 nm)/Mg: Ag. The maximum EQE, CE, and PE are 5.6%, 11.3 cd/A, and 7.1 lm/W. The EQE of the device reaches 3.3% at a brightness of 1000 lm/W, which has a rather low efficiency roll-off. It is one of the most efficient host-free blue OLED devices using traditional AIE fluorescent layers without any external coupling method [65,66,67].

**Figure 4:**
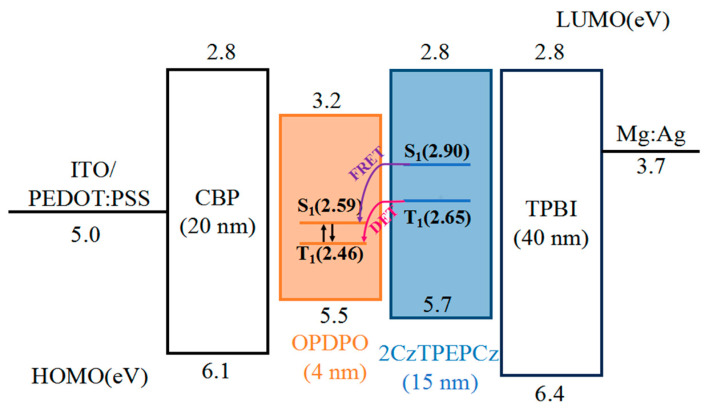
The energy diagram of the WOLED device and the diagram of the triplet harvesting mechanism.

It is shown that the TADF-fluorescent hybrid has the advantage of low rolling efficiency compared to all-TADF WOLEDs. Based on an important advantage of traditional fluorescent light-emitting layers, blue OLEDs generally have lower efficiency roll-off compared to yellow TADF OLEDs. Based on this, a completely host-free two-color WOLED has the following components: ITO/poly(3,4-methylenedioxy-thiophene): poly(styrene sulfonate) (PEDOT: PSS) (40 nm)/CBP (20 nm)/OPDPO (4 nm)/2CzTPEPCZ (15 nm)/TPBI (40 nm)/Mg: Ag with dual EMLs without any intermediate layer was constructed, and its structure and energy level diagrams are shown in Figure 4.

The tightly stacked light-emitting layer devices make charge carrier recombination more efficient into the 2CzTPEPCz layer from the TPBi light-emitting layer and then into the OPDPO light-emitting layer, where the energy barrier is avoided. It is also easy for holes to enter the OPDPO layer from the CBP luminescent layer. Due to the low HOMO energy level barrier, some holes enter the 2CzTPEPCz through the OPDPO luminescent layer. Most excitons compound in the OPDPO luminescent layer, and only a small part of them are at the 2CzTPEPCz interface, resulting in yellow light accounting for a greater proportion of the emission. OPDPO has a wide bandwidth, so the device ends up with warm white light. In summary, most excitons can be used for radioluminescence by timely or delayed fluorescence of OPDPO, thus obtaining higher EQE.

**Figure 5:**
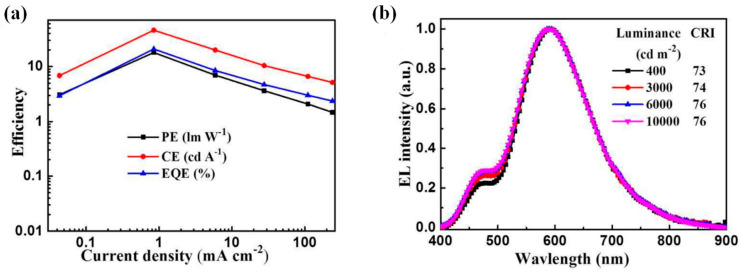
(**a**) PE-CE-EQE-current density curves and (**b**) normalized EL spectra at different luminance of the WOLED device.

From the characteristic curve in Figure 5, the maximum PE, CE, and EQE are 18.0 lm/W, 45.9 cd/A, and 20.8%. At a brightness of 1000 cd/m^2^, the EQE achieved by the device still reaches 13.7%, which is much higher than the EQE of most traditional host-free fluorescent WOLEDs reported in the previous literature. As the brightness increased from 400 to 10,000 cd/m^2^, the device CIE coordinates and CCT varied slightly from (0.45, 0.44) to (0.43, 0.44) and 3049 to 3392K, respectively, with the color rendering index (CRI) increasing from 73 to 76. A completely warm WOLED of the host-free two-color device, based on a traditional fluorescence-TADF hybrid emitter, has been successfully fabricated.

## 3. WOLEDs Based on Host-Free Phosphorescence-TADF Materials

Traditional fluorescent WOLEDs have limitations in achieving high efficiency, with only 25% maximum IQE [23]. However, the phosphorescent introduction of light-emitting layer molecules has significantly improved OLED efficiency [68,69]. These phosphorescent materials typically contain heavy metal atoms, such as Os, Pd, Pt, and Ir, in their organic metal complexes [70]. Compared to traditional luminescent materials, the singlet S_1_ and triplet T_1_ excitons can be effectively utilized by the phosphor through the heavy atom effect, resulting in a potential IQE of 100% and enabling the realization of high-performance OLEDs [71,72]. Furthermore, due to their high cost, fluorescence-based materials are less feasible for mass production, making phosphorescent materials the primary focus of experimental research [73,74]. In 2020, Huo et al. [75] demonstrated for the first time that bi-TADF-TADF /room-temperature phosphorescence (RTP) emission can be utilized to obtain WOLED with a high CRI.

**Figure 6:**
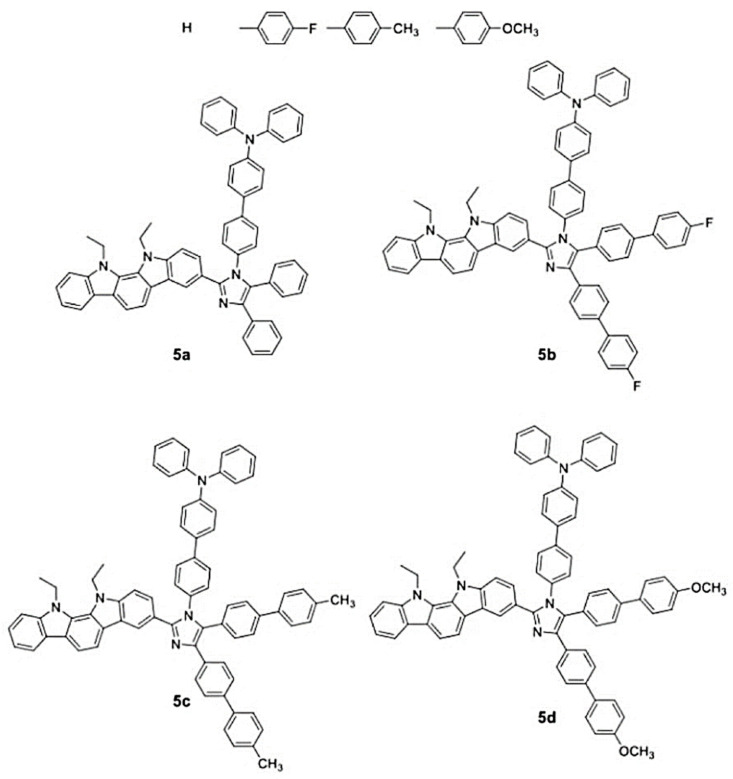
Molecular formula representation of the donor-acceptor-donor (D-A-D) compounds.

In 2022, Wu et al. [76] conducted a study in which a series of compounds with AIE properties were designed and synthesized. These compounds belong to the donor-acceptor-donor (D-A-D) type and utilize the donor-acceptor-donor’ (D-A-D′) framework, as depicted in Figure 6.

A high-efficiency WOLED was studied, in which the Irppy_3_ and Ir-(MDQ)_2_(acac)-doped compound was used as the double-blue-emission layer. The WOLED maximum brightness was 66,424 cd/m² and it had a CE of 67.6 cd/A. Notably, the device demonstrated excellent chromatic stability with minimal deviation (Δx = ±0.003, Δy = ±0.002) when the turn-on voltage changed from 5 V to 12 V. The color stability was improved by optimizing the structure of the double-blue-emission layer. High blue-fluorescence emission intensity was better matched with green and red phosphor emission intensity. The harmonious balance among the emission colors contributed to the device’s color stability and excellent electroluminescent properties. These findings underscore the significant potential of the newly developed wide band gap materials for applications in white lighting devices.

**Figure 7:**
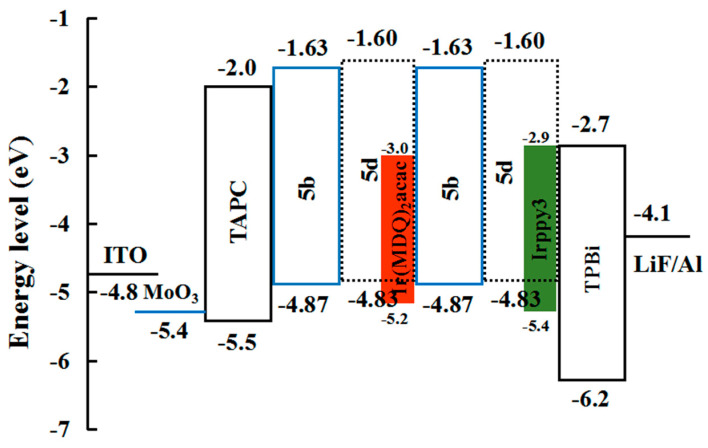
Schematic representation of the device structure for a hybrid WOLED.

Compound **5b**, used as a host-free blue OLED, exhibits excellent luminous performance with the 10,770 cd/m² maximum luminance and a CE of 6.93 cd/A. The device structure of the hybrid WOLED is illustrated in Figure 7. In this configuration, the fluorescent double emission layer uses compound 5b’s material, while compound **5d** is doped with Irppy_3_ (with an energy transfer (ET) value of 2.4 eV) and IR-(MDQ)_2_ACAC (with an ET value of 2.0 eV) to function as the green and red phosphorescent emission layers, respectively. The electron transport layer material uses TPBi. The hybrid WOLED demonstrated impressive performance metrics, including the 66,424 cd/m² maximum luminance, the 67.6 cd/A maximum CE, the 56.6 lm/W maximum PE, and the 24.3% maximum external quantum efficiency. The device was (0.337,0.341) in CIE coordinates, the CRI reached 85, and the pure white light emission was realized.

### 3.1. Phosphorescent Ultrathin Emitting Layer WOLED

**Figure 8:**
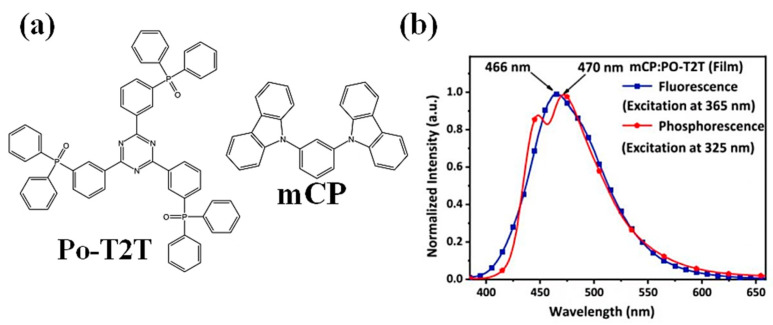
(**a**) Molecular structure formula representation; (**b**) low-temperature photoluminescence spectra of MCP:PO-T2T (1:1) films.

Although controlling ultrathin EML is more difficult to control in experiments than traditional host-free EML, it can avoid intramolecular aggregation, thereby building highly efficient host-free WOLED. In 2019, Li et al. [77] proposed a novel concept interlayer (IL) to insert sky-blue ultrathin emission layers (Ph-UEMLs) between the yellow and blue-light-emitting layers, effectively reducing the efficiency roll down of TADF. The device finally achieved 24.47% EQE. In 2023, Miao et al. [78] proposed a straightforward, host-free approach that uses single or complementary phosphorescent Ph-UEMLs directly in the interface polymers of TADF OLEDs. By implementing this method, all monochromatic OLEDs achieved the 2.4–2.5 V low turn-on voltage and exhibited remarkable device performance, with EQE max surpassing 20%. bis(4,6-difluorophenylpyridinato-N, C2)picolinate iridium (FIrpic) and bis(2-phenylbenzothiazolato)(acetylacetonate)iridium(III)(IR(BT_2_(ACAC))) were used as complementary Ph-UEMLs and directly stacked onto the interface of the m-bis(N-carbazolyl)benzene(mCP)/2,4,6-tris[3-(diphenylphosphine)phenyl]-1,3,5-triazine (PO-T2T)) exciton system. In order to study the TADF characteristics of exciton formation at the MCP/PO-T2T interface, the photoluminescence spectra of co-deposited MCP: PO-T2T films were studied at low temperatures, as depicted in Figure 8. The results indicate an energy level difference of 0.03 eV, satisfying the thermodynamic conditions for the RISC of triplet excitons. This finding suggests that the excitons formed within the mCP/PO-T2T system possess TADF characteristics.

**Figure 9:**
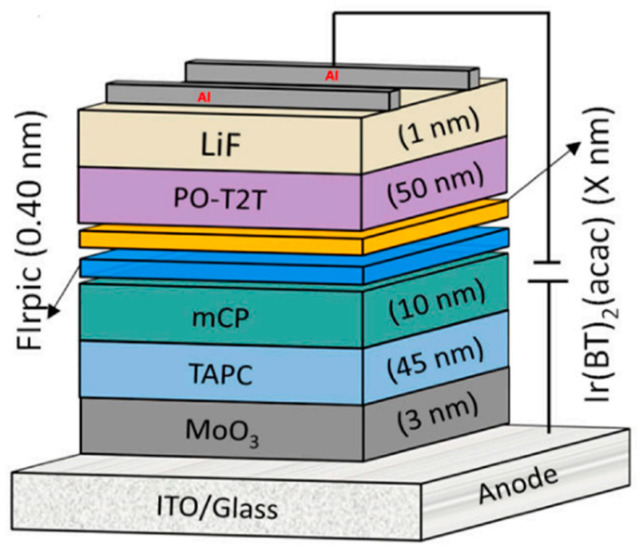
Schematic diagram of two-color white-light-emitting-layer device.

The optimized device structure consists of ITO (180 nm)/MoO_3_ (3 nm)/TAPC (40 nm)/mCP (10 nm)/FIrpic (0.40 nm)/Ir(BT)_2_(acac) (0.01, 0.02, 0.03, 0.04 nm)/PO-T2T (50 nm)/LiF (1 nm)/Al (100 nm), forming a two-color white-light-emitting-layer device, as illustrated in Figure 9. Remarkably, the device achieved a 23.48% maximum EQE, surpassing the EQE values of most doped dichroic white devices. Notably, the white light emission spectrum can be easily controlled from cold to warm by simply adjusting the thickness of the Ph-UEMLs.

In the experimental investigation, four devices were fabricated and tested, each incorporating phosphorescence. Ph-UEMLs with different thicknesses denoted as 0.01, 0.02, 0.03, and 0.04 nm correspond to the devices labeled as W1, W2, W3, and W4, respectively. Table 2 summarizes the key performance parameters of WOLED.

The effect of the thickness of IR(BT)_2_(ACAC)-UEML on the emission characteristics of WOLEDs has been studied systematically. As the IR(BT)_2_(ACAC)-UEML thickness increases, the relative intensity of blue emission decreases gradually, while that of yellow emission increases, eventually dominating the emission spectrum. At an IR(BT)_2_(ACAC)-UEML thickness of 0.02 nm, a well-balanced intensity between the two emission bands was achieved, resulting in the W2 device displaying a more balanced white light. By simply adjusting the PhUEML thickness, it is possible to achieve controlled white light emission with a tunable color temperature ranging from cold to warm. Remarkably, the W2 device demonstrated a 23.48% max EQE, surpassing most doped two-color WOLEDs, while maintaining the 2.5 V low turn-on voltage.

In order to obtain high-performance WOLEDs, the combination of TADF and phosphorescent materials has attracted great attention. However, the lack of stable and efficient blue phosphors has hindered the development of phosphorescent WOLEDs [79,80]. To address this challenge, various strategies have been proposed to achieve efficient white emission at the exciton scale [81,82]. Nonetheless, these device designs often introduce complexity and increase production costs. In light of this, a promising approach involves inserting an ultrathin phosphorescent emission layer into a blue fluorescent emissive layer, offering advantages such as simplified processing and flexible structural design. Zhao et al. [83] successfully implemented this approach by inserting a host-free ultrathin phosphorescent emission layer into a bipolar transport blue TADF emissive layer. The resulting TADF/phosphorescent hybrid WOLEDs achieved a 41.6 cd/A maximum CE, a 41.0 lm/W maximum PE, and a 19.1% maximum EQE.

### 3.2. Conventional Fluorescent and Phosphorescent Doped Hybrid WOLEDs

The main blue fluorescent materials used in WOLEDs are severely quenched due to the existence of too many singlet and triplet excitons [84,85,86]. In 2022, Zhang et al. [87] addressed this issue by incorporating the excellent bipolar TADF material, 1-(4-(4,6-Diphenyl-1,3,5-triazine-2-yl)phenyl)-1H-spiro[[pyridine-9,9′-fluorene (SpiroAC-TRZ), as the blue emitter. To enhance device efficiency and simplify the fabrication process while mitigating exciton quenching, an ultrathin orange phosphorescent emitting layer was inserted at a suitable position between the blue bipolar TADF emitters.

**Figure 10:**
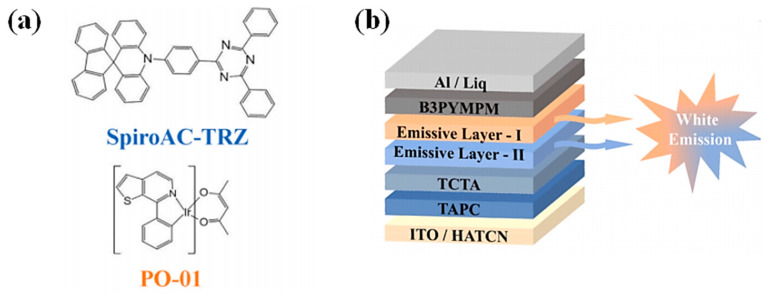
(**a**) Chemical structures of the emissive materials. (**b**) Structural representation of the hybrid WOLED.

The resulting WOLEDs exhibited an optimized structure of ITO/hexaazatriphenylenehexacabonitrile (HATCN)/1,1-bis[(di-4tolylamino)phenyl]cyclohexane (TAPC)/4,4′,4′′-Tri(9-carbamoyl) triphenylamine (TCTA)/SpiroAC-TRZ/bis(4-phenyl-thieno[3,2-c]pyridinato-C2,N)(acetylacetonate)iridium(III)(PO-01)/SpiroAC-TRZ/PO-01/SpiroAC-TRZ/PO-01/SpiroAC-TRZ/4,6-bis(3,5-di(pyridine-3-yl)phenyl)-2-(pyridine-3yl) pyrimidine (B_3_PYMPM)/Liq/Al, as depicted in Figure 10. At maximum luminance, the device achieved a 47.4 lm/W maximum PE, a 46.2 cd/A maximum CE, and a 15.0% maximum EQE. Notably, even at a brightness level of 1000 cd/m^2^, the device maintained a respectable PE of 41.7 lm/W, a CE of 45.8 cd/A, and an EQE of 14.7%. By incorporating the bipolar blue TADF material Spiro AC-TRZ and strategically inserting an ultrathin orange-phosphorescent-emitting layer, Zhang et al. successfully achieved efficient WOLEDs with a simplified, host-free device structure. These advancements not only reduced the efficiency roll-off but also mitigated exciton quenching, leading to improved overall device performance.

**Figure 11:**
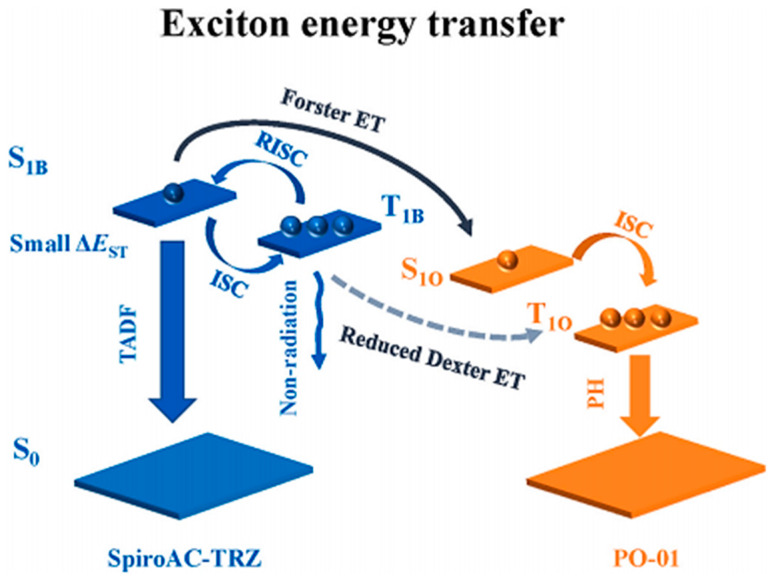
Exciton energy transfer schematic of SpiroAC-TRZ and PO-01.

The utilization of SpiroAC-TRZ as a bipolar transport material in WOLEDs not only benefits the device’s electrical characteristics but also contributes to improved exciton diffusion and reduced exciton confinement, thereby simplifying efficiency roll-off. SpiroAC-TRZ’s inherent bipolar transport properties enable efficient charge carrier migration in the device structure, resulting in a low turn-on voltage [83,88,89]. Furthermore, the incorporation of SpiroAC-TRZ as a blue TADF emissive layer facilitates balanced charge transport, expanded exciton distribution [90], reduced exciton quenching, and enhanced efficiency, increasing the device stability [91]. This is illustrated in Figure 10, which depicts the exciton energy transfer diagram between the blue TADF emissive layer and the orange phosphorescent emissive layer. Clearly, the SpiroAC-TRZ’s bipolar transport characteristics not only streamline vehicle transportation within the device but also contribute to low turn-on voltages. The incorporation of SpiroAC-TRZ as a blue TADF emissive layer enhances charge balance, broadens the exciton distribution area, reduces the depth of exciton confinement, and simplifies efficiency roll-off. The exciton energy transfer diagram presented in Figure 11 provides a visual representation of these phenomena.

## 4. WOLEDs Based on Host-Free Full-TADF Materials

Compared to WOLEDs based on conventional fluorescent materials or expensive phosphorescent materials containing heavy metals, all-TADF WOLEDs offer a promising alternative [92,93,94]. These all-TADF WOLEDs can achieve emission through RISC processes without the need for precious metals, making them cost-effective and environmentally friendly [95,96]. The combination of singlet and triplet excitons in TADF emitters yields high device efficiency at a lower cost. Both phosphorescence and TADF mechanisms have the potential to achieve 100% IQE in OLEDs [97,98]. However, the stability of phosphorescent materials due to their material characteristics remains a significant challenge [99,100]. Consequently, there is a growing interest in exploring all-TADF systems, although research in this area is still relatively limited.

**Figure 12:**
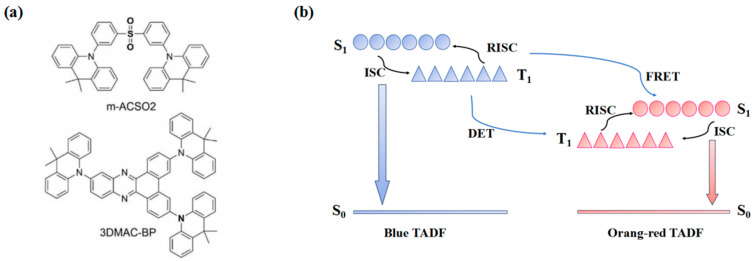
(**a**) Chemical structures of m-ACSO_2_ and 3DMAC-BP. (**b**) Schematic diagram of energy transfer between m-ACSO_2_ and 3DMAC-BP.

Existing all-TADF WOLED studies are not in depth. In 2019, Tang et al. [101] designed and synthesized bis[3-(9,9-dimethyl-9,10-dihydropyridine)phenyl]sulfone (m-ACSO_2_), which had a large triplet energy (T_1_ = 2.94 eV), a tiny energy gap, and a photoluminescence quantum yield (PLQY) up to 76% in neat films. The synthesized 3,6,11-tris(9,9-dimethylacridin-10(9H)-yl)dibenzo[a,c]phenazine (3DMAC-BP) orange TADF material, with a 0.012 eV single-wire triple-state energy gap and an 89% PLQY and other excellent TADF characteristics, is shown in Figure 12a. The emission peaks of ACSO_2_ and 3DMAC-BP were at 478 nm and 605 nm. Since m-ACSO_2_ and m-ACSO_2_ are in close contact at the ultrathin interface, the triplet energy of m-ACSO_2_ (T_1_ = 2.94 eV) is larger than 3DMACBP (T_1_ = 1.98 ev), the triplet energy transfer (DET) between the two is easy to achieve. Figure 12b shows the exciton energy transfer process between m-ACSO_2_ and 3DMAC-BP.

The host-free WOLEDs with full TADF were prepared as follows: ITO/PEDOT: PSS (60 nm)/m-ACSO_2_ (40 nm)/3DMAC-BP (0.15 nm)/m-ACSO_2_ (x = 0, 10, 20 and 30 nm)/TmPyPB (50 nm)/LiF (1 nm)/Al (120 nm). When X = 20, the device turn-on voltage is 2.6 eV, and bimodal white emission is achieved. In addition, at the 1000 cd/m^2^ luminance, the device exhibits good warm white emission with International Lighting Committee CIE coordinates of (0.34, 0.43). When the voltage rises from 5 V to 8 V, the EL spectrum also shows a small change, and the device CRI value remains above 74. By controlling the position of the 3DMAC-BP ultrathin layer and comparing its different EL spectra, it can be proven that the orange–red ultrathin layer position controls the emission spectrum by controlling the distribution of excitons in the sky-blue-light-emitting layer. The results show that the thickness of 0.15 nm and x = 10’s device has the best performance, has a 51.7 cd/a maximum CE, a 46.4 lm/W maximum PE, and a 24.2% maximum EQE. The device simplifies the device structure, reduces manufacturing complexity, and finally achieves high efficiency through the form of an ultrathin layer.

In a separate study, Zhong et al. [102] designed and synthesized 9,9-dimethyl-10-phenyl-acridan (BDMAc) as a deep blue luminescent layer in 2019. By incorporating 2-(10H-phenothiazin-10-yl)dibenzothiophene-S, S-dioxide (PVTZ-DBto2) as the yellow-emitting layer and host material in conjunction with BDMAc, they achieved a maximum EQE of 14.2%. Importantly, the construction of fully TADF-based single emission layer (SEL)-WOLEDs had led to significant advancements in achieving color-balanced white emission. These devices exhibited impressive performance characteristics, including high EQE, CE, and PE values. Notably, the incorporation of TADF material, such as 2,3,4,5,6-pentakis(3,6-dimethyl-9H-carbazol-9-yl)benzonitrile (5PCzCN), had demonstrated exceptional stability, with extended lifetimes at various brightness levels. Additionally, the design and synthesis of novel luminescent materials, such as 9,9-dimethyl-10-(3-vinylphenyl)-9,10-dihydropyridine (BDMAc), in combination with suitable hosts, had contributed to achieving high EQE values in specific color regions.

**Figure 13:**
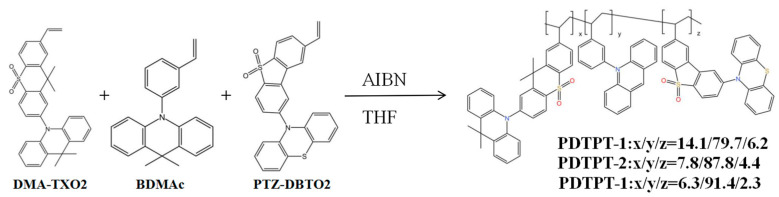
Molecular structures of polymers synthesized using DMA-TXO_2_, BDMAc, and PVTZ-DBTO_2_.

**Figure 14:**
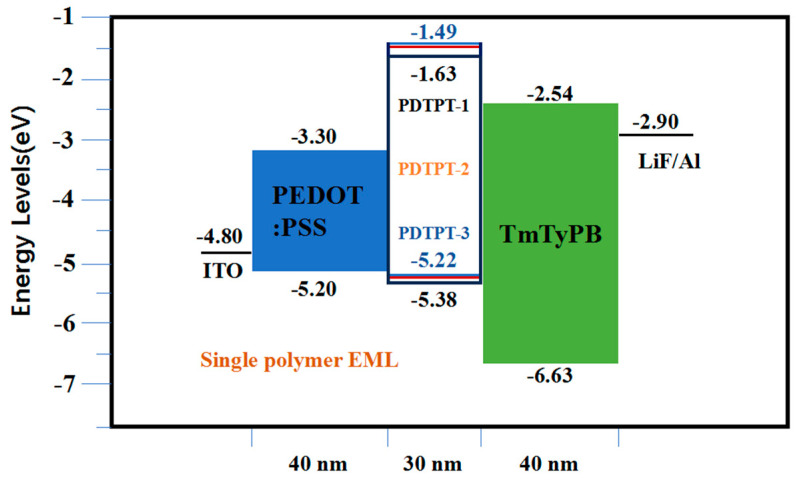
Schematic of the device structure diagram for a two-layer device configuration.

Figure 13 illustrates the polymers obtained through radical copolymerization of 2-(9,9-dimethy-acridine-10-yl)-8-vinyl dimethyl thioxanthene-S, S-dioxide (DMA-TXO_2_), BDMAc, and 2-(10H-phenothiazine-10-yl)-8-vinyl dibenzothiophene-S, S-dioxide (PTZ-DBTO_2_) monomers. The resulting polymers exhibit a mean RMS roughness within the narrow range, indicating a remarkably smooth and uniform film morphology. This suggests that these polymers possess desirable characteristics as EML materials for OLEDs. To optimize the device performance, a dual-layer device structure consisting of ITO/ PEDOT: PSS (40 nm)/EML (30 nm)/TmPyPB (40 nm)/LiF (1 nm)/Al (100 nm) was employed, as depicted in Figure 14.

Notably, the radical copolymerization of DMA-TXO_2_, BDMAc, and PTZ-DBTO_2_ monomers yielded polymers with highly smooth and uniform film morphologies. These polymers hold great potential as EML materials in OLEDs. The optimized device structure, incorporating these polymers as the emitting layer, follows a dual-layer architecture and demonstrates promising prospects for achieving enhanced OLED performance.

The PDTPT-1-based device exhibited notable characteristics, including a 2900 cd/m^2^ maximum brightness, a 38.8 cd/A maximum CE, a 20.3 lm/W maximum PE, and a (0.33, 0.42) CIE coordinates. The trap effect in the device arises from the host and adjacent layers having a significant energy gap [103], resulting in a large energy barrier for charge injection into the host layer. Additionally, the emitter possesses a deeper Lowest Unoccupied Molecular Orbital (LUMO) level and a shallower Highest Occupied Molecular Orbital (HUMO) level compared to the host, enabling direct charge injection and subsequent radiative decay in the emissive layer [104,105]. Notably, the HOMO level of BDMAc, an emitter in the optimized device, closely aligns with both the two emitters and the HOMO level of PEDOT: PSS, minimizing the energy barrier. In contrast, a substantial LUMO gap exists between BDMAc and the adjacent layer TmPyPB, while TMPYPB and DMA-TXO_2_ or PGTZ-DBTO_2_ exhibit only a small 0.1 eV barrier. Consequently, direct charge injection into the emissive layer is facilitated. The device offers several advantages:

1. The high T_1_ and shallow HOMO level of BDMAC prevent triple energy back transfer (TEBT) from the blue and yellow emitters to the host. Moreover, it provides a minimal energy barrier for hole injection from the ITO/PEDOT:PSS anode into the polymer.

2. In comparison to DMA-TXO_2_, DMA-TXO_2_ retains 90% of the PLQY and the triple levels of 3.08 eV. However, the HOMO energy of DMA-TXO_2_ is shifted from −6.10 eV to −5.50 eV, enabling better compatibility with the host.

3. The close physical contact of the PDTPT-1 intermolecular chains enhances the Förster resonance energy transfer (FRET) and DET processes, leading to efficient energy transfer. The trap-assisted recombination and Langevin recombination mechanisms ensure a balanced charge flux and a wide recombination zone, thereby reducing the formation of high-energy excitons on the host and minimizing exciton quenching. The utilization of dual-channel recombination contributes to enhanced device efficiency.

Clearly, the PDTPT-1 based device clearly demonstrates favorable characteristics, including high brightness, CE, and PE values. The trap effect, combined with optimized energy levels and intermolecular interactions, allows for efficient charge injection, reduced exciton quenching, and improved device performance.

## 5. Conclusions

In summary, this review discusses the performance of WOLEDs based on TADF in various configurations, including TADF-phosphorescence, TADF-fluorescence, and all-TADF devices. Among the different configurations, the efficiency of TADF-phosphorescent devices reaches 20.8%, while having good color stability and a high CRI [63]. The brightness of TADF-fluorescent devices is as high as 66,424 cd/m² while the CE of 67.6 cd/A is reached [76]. The host-free WOLED device composed of all TADF materials has the highest efficiency, reaching 24.2% EQE [101]. The TADF-phosphorescent host-free device reached 23.48%, with a low turn-on voltage of 2.5 V. By adjusting the thickness of the emitting layer, this device could emit white light with a tunable color temperature ranging from warm to cold. The all-TADF devices achieved superior efficiency, CE, and PE compared to solution-treated mixed WOLEDs. In some cases, they even approached the performance of all-phosphorescent devices. Notably, the host-free full-fluorescence optimized device achieved a record 130.7 lm/W PE and 31.1% EQE.

Although all phosphorescent WOLED structures can achieve high efficiency, but the high cost, low stability, and blue phosphorescent emitters remain significant challenges for large-scale applications. To solve this problem, researchers have explored the integration of complementary fluorescence or phosphorescence emitters with TADF in WOLEDs. Additionally, the solution treatment method has been investigated as an alternative to traditional thermal evaporation techniques, effectively addressing the dissolution issue between adjacent functional layers. However, further research is needed to optimize film design based on material and device requirements.

Over nearly three decades of research, WOLEDs have achieved remarkable advancements in operational longevity, luminous efficiency, device stability, and CRI. Fluorescent devices have demonstrated higher stability compared to all-TADF white devices. Nevertheless, all-TADF devices have rapidly advanced and now exhibit performance parameters comparable to phosphorescent WOLEDs, which have reached commercial viability. While phosphorescent materials remain costly, TADF-based white light devices offer promising alternatives. Despite current limitations in terms of cost, lifetime, and efficiency, considering the widespread adoption of OLED technology in displays and lighting, we can foresee continuous advancements in theoretical understanding, process development, and material research for WOLEDs. These advancements will help overcome technological barriers and ultimately benefit all of humanity.

## Figures and Tables

**Table 1:** Device performance of TADF based WOLEDs.

Devices	CE (cd/A)	PE (lm/w)	CRI	CIE (x,y)	EQE (%)	Ref.
TADF Ultrathin WOLED	30.3	28.8	88	(0.47,0.44)	18.7	[39]
	34.14	33	−	(0.369,0.460)	−	[40]
	18.9	21.1	86	(0.375,0.410)	8.7	[41]
TADF Doped WOLED	28.83	20.83	−	(0.45,0.46)	11.5	[42]
	16.3	13.3	−	(0.37,0.39)	8.39	[35]
	26.04	23.83	90	(0.490,0.473)	12.79	[43]
TADF-phosphor sensitization WOLED	60.0	42.8	64	(0.33,0.44)	22.8	[36]
	46.8	56.5	−	(0.44,0.48)	15.2	[44]
	26.9	22.3	−	(0.51,0.41)	12.8	[45]
TADF interfacial exciplex WOLED	52.8	33.2	80	(0.35,0.39)	22.9	[46]
	69	69.9	−	(0.40,0.57)	20	[47]
	57.8	62.5	−	−	22.7	[48]

**Table 2:** Key performance parameters of white components W1, W2, W3, and W4.

Devices	Turn-On Voltage (v)	Maximum			
		CE (cd/A)	PE (lm/W)	EQE (%)	Luminance (cd/m^2^)
W1	2.5	53.96	64.77	20.50	12,140
W2	2.5	64.93	78.80	23.48	13,220
W3	2.5	59.96	75.35	21.27	16,050
W4	2.5	57.50	71.02	20.29	16,870
